# The therapeutic use of 177 Lu-PSMA-617 radioligand therapy in prostate cancer treatment: a review of literature and ongoing trials

**DOI:** 10.1007/s12672-024-01680-z

**Published:** 2024-12-18

**Authors:** Alexander Konopnicki, Michael Zaliznyak, Mathews Roy, Bagi Jana

**Affiliations:** 1 Department of Internal Medicine, University of Texas Medical Branch, Galveston, TX USA; 2 Department of Urology, University of California San Francisco, San Francisco, CA USA; 3 Department of General Oncology, The University of Texas MD Anderson Cancer Center, Houston, TX 77030 USA

**Keywords:** Prostate Cancer, ^177^Lu-PSMA-617, Radioligand therapy

## Abstract

Radioligand therapy is a targeted cancer treatment modality in which radioisotopes are utilized in the delivery of radiation at targeted cancer cells, with the goal of sparing normal cells. Prostate cancer is a well-known radiosensitive disease, historically treated with radioisotopes such as Strontium-89, Samarium-153, and Radium-223 for palliation of bone metastases. Recently, prostate specific membrane antigen (PSMA) has recently been employed as a radioligand target due to its unique properties of high expression on the surface of prostate cancer cells, limited expression in normal tissue, function as an internalizing cell surface receptor, and increased expression with androgen deprivation therapy. In 2015, ^177^Lu-PSMA-617 was first introduced as a promising treatment option for castration-resistant prostate cancer, and 7 years later the results of the phase III VISION trial led to ^177^Lu-PSMA-617 gaining FDA approval for the treatment of progressive castration-resistant prostate cancer. These results in combination with the inherent properties of ^177^Lu-PSMA-617 have led to its current exploration as a promising treatment modality beyond progressive metastatic castration-resistant prostate cancer, and into the earlier phases of prostate cancer. This review paper aims to highlight the key phase III randomized controlled trials related to ^177^Lu-PSMA-617 in all stages of prostate cancer, as well as bring attention to ongoing, earlier phase I/II trials incorporating ^177^Lu-PSMA-617.

## Introduction

Theranostics radioligand therapy (RLT) refers to the use of a pair of radiopharmaceuticals containing radionuclides for imaging and therapy [1]. It allows for a targeted cancer treatment modality in which radioisotopes are utilized in the delivery of radiation at targeted cancer cells, with the goal of sparing normal cells [2]. The concept was first used by Dr. Saul Hertz in 1941 for the treatment of thyroid disease with radioactive iodine [3]. Since then, RLT has emerged as a promising treatment modality in a variety of radiosensitive diseases. One such radiosensitive disease is prostate cancer, in which RLT has been studied in both localized and metastatic disease states. The beta-emitting radioisotopes, Strontium-89 and Samarium-153, were among the first radioisotopes approved for palliation of painful bony metastases. Strontium-89 was first approved in 1993, while Samarium-153 was approved in 1997 [4, 5]. The alpha-emitting radioisotope, Radium-223, was more recently approved for treatment of symptomatic bone metastases in castration-resistant prostate cancer (CRPC) in 2013 [6]. However, these radioisotopes lacked selectivity for target tissue and have been found to non-selectively bind to areas of increased bone metabolism. The first FDA-approved RLT used peptide receptor radionuclide therapy for progressive midgut neuroendocrine tumors in 2018. This combined the radionuclide ^177^Lu with the somatostatin analogue DOTATATE to deliver ionizing radiation to tumor cells expressing somatostatin receptors [7]. Now, prostate specific membrane antigen (PSMA) has become an area of interest for targeted radionuclide therapy that is highly-specific for the target tumor tissue.

PSMA, or folate hydrolase I and glutamate carboxypeptidase II, is a cell membrane protein that is expressed on the surface of prostate cancer cells [8]. It has emerged as a beneficial radioligand target in prostate cancer treatment due to its limited expression in normal tissue, high expression levels on the plasma membrane of the tumor cell, function as an internalizing cell surface receptor [9], and increased expression with androgen deprivation therapy (ADT) [10]. In 2015, ^177^Lu-PSMA-617 was first introduced as a promising modality for CRPC [11], and 7 years later the results of the phase III VISION trial led to ^177^Lu-PSMA-617 emerging as a potentially favorable treatment modality in earlier stages of prostatic disease [12].

Despite these advancements, prostate cancer remains a highly prevalent disease, and one that will likely see a continued growth in incidence due to an increasingly aging global population. This calls attention to the importance of highlighting the literature supporting the advancements in modern treatments. The purpose of this narrative review is to describe the current body of evidence supporting the use of ^177^Lu-PSMA-617 in prostate cancer. The results of recent large cohort trials in combination with the inherent properties of ^177^Lu-PSMA-617 have led to its current exploration as a promising treatment modality beyond progressive metastatic CRPC (mCRPC), and into the earlier phases of prostate cancer treatment.

### Prostate cancer and standard of care treatment

Prostate cancer is the second leading cause of male cancer-related mortality in the United States, with an estimated 288,300 new cases and 34,700 deaths in 2023 [13]. Early detection and management of low to intermediate risk prostate cancer is associated with favorable outcomes including prostate cancer-specific survival at 5 and 10-years approaching 100% [14]. However, the disease becomes more difficult to manage in patients with high-risk disease or evidence of metastasis [15], with approximately one third of all prostate cancer patients developing biochemically recurrent disease after primary definitive therapy [16].

In addition to being a radioligand target, PSMA is also an effective imaging marker. PSMA-directed PET is a non-invasive diagnostic technique to image PSMA positive lesions in individuals with prostate cancer. The routine clinical uses of PSMA PET allows for initial staging of prostate cancer as well as evaluation of metastasis and disease recurrence. Patients who are found to have disease recurrence confirmed on PSMA PET or biochemical recurrence on PSA surveillance, are subsequently offered a variety of treatment options which include hormonal therapy, radiation therapy, or immunotherapy.

Since the 1940s, ADT has been the backbone of prostate cancer treatment for patients with biochemical recurrent disease [17]. The initiation of ADT marks the beginning of treatment for castration-naive prostate cancer (CNPC) [18], with the addition of an androgen receptor pathway inhibitor (ARPI) for patients with metastatic castration naive prostate cancer (mCNPC). Examples of ADT include GnRH agonists such as leuprolide, goserelin, triptorelin, and buserelin. Commonly prescribed treatments for mCNPC are the combination of abiraterone, a first-generation ARPI, and prednisone to ADT [19–21], and the second-generation ARPIs, enzalutamide, apalutamide, and darolutamide [22–24]. However, despite these regimented therapies, a subset of these patients will continue to exhibit disease progression at which point their disease state becomes known as CRPC.

The treatment of CRPC is vast and varies depending on the presence of metastasis. Nonmetastatic disease is treated with continued ADT and an ARPI such as enzalutamide, apalutamide, and darolutamide [25–27]. For metastatic disease without prior ARPI exposure, treatment is augmented by the addition of abiraterone plus prednisone [28, 29] or enzalutamide [25, 30]. Docetaxel plus prednisone is the standard of care for initial chemotherapy in males with relatively rapidly progressing symptomatic mCRPC [31], with cabazitaxel plus prednisone serving as the preferred next-line agent for patients who progressed on treatment with docetaxel and required additional chemotherapy [32]. While cabazitaxel offered a survival benefit in men with taxane-refractory mCRPC relative to current treatment regimens, that survival benefit was only 2.4 months [33, 34].

### Key studies: phase III randomized controlled trials

#### Progressive castration-resistant prostate cancer

In 2015, ^177^Lu-PSMA-617 RLT was first introduced as a promising treatment modality for mCRPC [11]. This therapy gained popularity due to its favorable properties, including a long half-life and optimal medium energy. A long half-life is favorable due to a sustained treatment effect, dosing flexibility, and waste minimization. Medium energy is optimal because of targeted cell destruction with minimal damage to the surrounding tissue, and an effective range to balance systemic side effects with effective treatment of the tumor cells. Several subsequent trials reported positive outcomes with treatment including reduced pain, low toxicity, and favorable response rates compared to standard therapy alone [34–38].

In 2021, the results of the TheraP trial, the first randomized study of ^177^Lu-PSMA-617 RLT, was published [34]. TheraP was a multicenter, unblinded, randomized phase 2 trial in which 200 patients with mCRPC and prior docetaxel treatment were 1:1 randomized to receive either ^177^Lu-PSMA-617 (6·0–8·5 GBq intravenously every 6 weeks for up to six cycles) or cabazitaxel (20 mg/m^2^ intravenously every 3 weeks for up to ten cycles). The primary endpoint of this trial was PSA response, defined by a reduction of over 50% from baseline. Secondary endpoints included radiographic progression-free survival (rPFS), adverse events, and death.

TheraP reported compelling evidence in favor of ^177^Lu-PSMA-617 as an active treatment for men with mCRPC who have progressed after docetaxel chemotherapy. The trial reported a more frequent PSA responses among men in the ^177^Lu-PSMA-617 group than in the cabazitaxel group (66% vs 37% by intention to treat; 95% CI 16–42; p < 0·0001; and 66% vs 44% by treatment received; [9–37]; p = 0·0016). Key secondary endpoints also favored the study arm. Progression-free survival (PFS) was significantly improved in the ^177^Lu-PSMA-617 group (HR, 0.63; 95% CI 0.46–0.86; p = 0.0028). Additionally, grade 3–4 adverse events were less frequent in the ^177^Lu-PSMA-617 group (33% vs 53%). Overall survival (OS) was similar among both groups (19.1 vs 19.6 months; HR, 0.97; 95% CI 0.70–1.4; p = 0.99), and no deaths were attributed to ^177^Lu-PSMA-617 therapy [34].

In 2022, the efficacy of ^177^Lu-PSMA-617 RLT in mCRPC was further expanded upon with the publishing of the phase 3 VISION trial which described the largest assessment of ^177^Lu-PSMA-617 RLT among mCRPC patients to date [12]. A total of 831 patients with mCRPC previously treated with at least one ARPI and one or two taxane regimens were randomly assigned, in a 2:1 ratio, to receive either ^177^Lu-PSMA-617 (therapeutic dose of 7.4 GBq for 6 cycles every 6 weeks) in addition to standard care (study group) versus receiving standard care alone (control group). The primary end points of this trial included rPFS and OS. Key secondary end points were objective response, disease control, and time to symptomatic skeletal events (SSE).

VISION reported significant improvement in patient outcomes among the study group compared with the control group. Compared with the control group, patients assigned to the study group demonstrated significantly prolonged rPFS (median, 8.7 vs. 3.4 months; hazard ratio for progression or death, 0.40; 99.2% CI 0.29 to 0.57; P < 0.001) and OS (median, 15.3 vs. 11.3 months; hazard ratio for death, 0.62; 95% CI 0.52 to 0.74; P < 0.001). Key secondary endpoints also favored the study group. The median time to the first SSE or death was 11.5 months in the study group, as compared with 6.8 months in the control group (hazard ratio, 0.50; 95% CI, 0.40 to 0.62; P < 0.001). Among patients who had measurable target lesions, a complete response was noted in 9.2% in the study group and in none of the patients in the control group. A partial response was noted in 41.8% in the study group versus 3% in the control group. Additionally, the proportion of patients with confirmed decreases in the PSA level of at least 50% was 46.0% in the study group compared to 7.1% in the control group (OR 11.19 [95% CI 6.25, 20.04]), while the decrease in PSA level of at least 80% was 33.0% in the study group compared to 2.0% in the control group (OR 23.62 [95% CI 8.57, 65.11]) [12].

The results of the VISION trial, as well as the culmination of efforts of previously reported literature [34–37], ultimately led to the FDA approval of ^177^Lu-PSMA-617 RLT for the treatment of patients with mCRPC in 2022 [39]. The results of these trials demonstrate a significant improvement in patient outcomes and suggests a positive step towards disease control in patients with mCRPC.

#### Taxane-naive metastatic castration-resistant disease

The phase 3 VISION trial showed ^177^Lu-PSMA-617 significantly prolonged rPFS and OS in patients with mCRPC previously treated with one or more ARPIs and 1–2 taxanes. However, the utilization of ^177^Lu-PSMA-617 in patients who are taxane-naive had not been previously described. This is being explored by the PSMAfore trial, which has been investigating the outcomes in taxane-naive patients with mCRPC treated with either ^177^Lu-PSMA-617 or a change in ARPI.

PSMAfore is a multicenter, open-label, randomized phase 3 trial in adults with progressive mCRPC and confirmed PSMA expression by ^68^ Ga-PSMA-11 PET/CT. In order to be eligible for this trial, patients had to have been taxane-naive in the metastatic setting with a history of receiving one prior ARPI, and who are candidates for a change in ARPI. The trial enrolled 469 patients who were randomized 1:1 to receive ^177^Lu-PSMA-617 (7.4 GBq IV every 6 weeks for 6 cycles) or a change in ARPI to either abiraterone or enzalutamide. The primary endpoint is rPFS. The key secondary endpoint is OS, as well as safety and tolerability of ^177^Lu-PSMA-617 and health-related quality of life [40].

Preliminary results were presented in October 2023 and showed the trial met its primary endpoint with a statistically significant benefit in rPFS, with a 59% reduction in the risk of radiographic progression in patients treated with ^177^Lu-PSMA-617 versus those who received a change in ARPI. The study was also statistically significant for several secondary endpoints, with patients in the experimental arm showing improved quality of life and delay in worsening pain. Patients in the experimental arm were also more than 2.5 times more likely to demonstrate a PSA decline of at least 50% compared with those treated with a change in ARPI alone [41]. Eighty-four percent of patients with progressive disease with ARPI alone chose to cross-over to the experimental arm.

Additional data was presented in September 2024, which further illustrated ^177^Lu-PSMA-617 prolonged rPFS relative to ARPI change, with a favorable safety profile. The median rPFS was 11.60 months (95% CI 9.30–14.19) in the ^177^Lu-PSMA-617 group versus 5.59 months (95% CI 4.21–5.95) in the ARPI change group (HR 0.49 [95% CI; 0.39–0.61]). The incidence of grade 3–5 adverse events was also lower in the ^177^Lu-PSMA-617 group compared to the ARPI change group [42].

The study is estimated to be completed in September 2025. The preliminary results of PSMAfore are encouraging and show the potential of ^177^Lu-PSMA-617 in improving patient outcomes in taxane-naïve patients with progressive mCRPC.

#### Castration-naive prostate cancer

The results of the VISION trial provided encouragement for studying the use of ^177^Lu-PSMA-617 beyond CRPC and into castration-naive disease. It has previously been described that PSMA expression occurs universally both prior to hormone deprivation and in the recurrent disease setting [43–45]. Additionally, studies have shown that radio-sensitization occurs with ADT and confers improved OS, local control, disease progression, and distant metastases free survival in comparison to treatments with radiotherapy alone [46, 47]. Following radiation therapy, the androgen receptor signaling pathway repairs the DNA damage caused by radiation. Blocking this pathway with ADT inhibits this repair mechanism, further exposing the cancer cells to radiation therapy [48, 49]. It is hypothesized that with the inhibition of these DNA repair mechanisms through ADT, there will be fewer genomic alterations that could lead to radiation-resistance in mCNPC. These results are being studied in the ongoing PSMAddition trial.

PSMAddition is an international, prospective, open-label, randomized, phase III trial comparing ^177^Lu-PSMA-617 in combination with standard of care, versus standard of care alone, in adult male patients with mCNPC. Approximately 1126 patients will be randomized 1:1 to receive ^177^Lu-PSMA-617 (7.4 GBq every 6 weeks for a maximum of 6 cycles) plus standard of care or standard of care alone. Standard of care is defined as a combination of ARPI plus ADT. The primary endpoint is rPFS. Upon confirmed radiographic progression, participants in the standard of care cohort can cross over to the ^177^Lu-PSMA-617 cohort. The key secondary endpoints include OS, proportion of patients with a PSA decline of ≥ 90% from baseline, time to development of mCRPC, composite PFS, safety, tolerability, and health-related quality of life [50]. As of May 2023, 176 centers are recruiting patients to the study, with an estimated study completion date in February 2026.

#### Oligometastatic prostate cancer

Oligometastatic prostate cancer (OMPC) refers to an intermediate disease state, between localized disease and widespread metastasis, in which patients are observed to have a limited number of sites of distant metastasis while still being a potentially curable disease [16, 51]. There is not an established number of quantifiable distant sites that define OMPC. Ongoing prospective studies define oligometastases as ranging from 1–5 sites of metastases [52, 53]. OMPC has classically been defined through conventional imaging (CT/MRI and bone scans). However, with the advent of PSMA-based imaging, we are now able to detect previously undetectable cases of OMPC, with PSMA PET/CT now being considered the most accurate diagnostic imaging modality for detecting oligometastasis in prostate cancer patients [54, 55]. Currently stereotactic body radiation therapy (SBRT) is considered the standard of care for OMPC [56], with the goal of delaying initiation of ADT and prolonging PFS [57, 58].

In March 2024, the PSMA-DC study was launched with the goal of evaluating the efficacy of ^177^Lu-PSMA-617 versus observation after SBRT in delaying castration or disease recurrence in adult patients with PSMA positive OMPC. PSMA-DC is an international, prospective, open-label, multi-center, randomized phase III study. Approximately 450 patients will be enrolled. Following SBRT, participants will be randomized in a 2:1 ratio to receive either ^177^Lu-PSMA-617 (7.4 GBq (200 mCi) ± 10% every 6 weeks for a planned 4 cycles) or observation only (control arm). The primary endpoint of PSMA-DC is to evaluate metastasis free survival (MFS). Key secondary endpoints include time to hormonal therapy, time to PSA progression, time to symptomatic progression, rPFS, OS, as well as safety, tolerability, and health-related quality of life. The estimated study completion date is July 2030 [59].

A summary of the key phase III randomized controlled trials (RCTs) related to ^177^Lu-PSMA-617 use in the various phases of prostate cancer can be found in Table 1.

**Table 1:** Summary of the Key RCTs Studying PSMA RLT in Prostate Cancer

Trial Name	Treatment Phase	Participants	Intervention Comparison (vs. ^177^Lu-PSMA-617)	Primary Endpoints	Secondary Endpoints	Results	Study Completion	Supporting Studies
TheraP	Progressive CRPC	200	Cabazitaxel	PSA Response	PFS; AEs; OS	PSA Response: 66% vs. 37% PFS: HR 0.63 AEs: 33% vs 53% grade 3–4 AEs OS: 19.1 vs 19.6 months	Yes 2021	Hofman et al
Vision	Progressive CRPC	831	Cabazitaxel	rPFS; OS	Objective response; time to first SSE	rPFS: 8.7 vs. 3.4 months OS: 15.3 vs. 11.3 months Time to first SSE: 11.5 vs 6.8 months Objective response: 9.2% vs none complete response; 41.8% vs 3% partial response	Yes 2022	Sartor et al
PSMAddition	mCNPC	1126	ARPI + ADT	rPFS	OS; proportion of patients with PSA decline ≥ 90% from baseline; time to development of mCRPC; composite PFS; safety, tolerability, and health-related quality of life	N/A	February 2026	Tagawa et al
PSMA-DC	Oligometastatic	450	Observation following SBRT	MFS	Time to hormonal therapy; time to PSA progression; time to symptomatic progression; rPFS; OS; safety, tolerability, and health-realted quality of life	N/A	July 2030	NCT05939414
PSMAfore	Taxane-Naïve Progressive CRPC	450	ARPI Change to Abiraterone or Enzalutamide	rPFS	OS; safety and tolerability; health-related quality of life	rPFS: 11.60 vs 5.59 months; lower incidence of grades 3–5 AEs	Preliminary results released September 2024; Projected September 2025	Sartor et al

RCT, randomized controlled trial; PSMA, prostate specific membrane antigen; RLT, radioligand therapy; CRPC, castration-resistant prostate cancer; mCNPC, metastatic castration-naïve prostate cancer; ARPI, androgen receptor pathway inhibitor; ADT, androgen deprivation therapy; SBRT, stereotactic body radiation therapy; PSA, prostate specific antigen; rPFS, radiographic progression-free survival; OS, overall survival; MFS, metastasis-free survival; ITT, intention to treat; AE, adverse events; SSE, symptomatic skeletal events

### Phase I/II ^177^Lu-PSMA-617 trials

In addition to the large phase III RCTs, there are several earlier phase clinical trials investigating the use of ^177^Lu-PSMA-617 in the various stages of prostate cancer disease states. We performed a review of clinicaltrial.gov using the keywords “Lu-177 PSMA-617” and “Prostate Cancer” and summarized the ongoing clinical trials herein. A summary of the following studies can be found in Table 2.

**Table 2:** Summary of the Phase I/II Trials Studying PSMA RLT in Prostate Cancer

Trial Name	Treatment Phase	Participants	Intervention (vs ^177^Lu-PSMA-617)	Primary Endpoints	Results
NCT03805594	mCRPC	43	Pembrolizumab	RP2D, schedule, ORR	N/A
NCT05766371	mCRPC	45	Pembrolizumab	Safety, clinical benefit of single dose	N/A
NCT05150236 (EVOLUTION)	mCRPC	110	Ipilimumab; nivolumab	PSA-PFS	N/A
NCT05113537 (UPLIFT)	mCRPC	30	Abemaciclib	DLTs; RP2D	N/A
NCT05613894 (CaboLu)	mCRPC	24	Cabozantinib	DLTs; MTD; recommended dose and schedule	N/A
NCT03874884 (LuPARP)	mCRPC	52	Olaparib	Safety and tolerability	N/A
NCT04419402 (ENZA-p)	mCRPC	162	Enzalutamide	PFS	13.0 vs 7.8 months
NCT05340374 (LuCaB)	mCRPC	44	Cabazitaxel	MTD; DLTs; RP2D	N/A
NCT06004661	mCRPC	20	Normal, moderately reduced, or severely reduced renal impairment	Effects of renal radiation absorption; radiation concentration in blood; urine radioactivity	N/A
REALITY	mCRPC	254	N/A	Changes from baseline PSA concentration; PSA-PFS; OS	≥ 50% PSA reduction in 52% of patients PSA-PFS: 5.5 months OS: 14.5 months
NCT04430192 (LuTectomy)	Localized Prostate Cancer	20	N/A	Radiation absorbed dose in prostate and lymph nodes; dosimetry, safety, and efficacy	Prostate radiation absorbed dose: 19.6 Gy Pelvic LN radiation absorbed dose: 37.9 Gy Safe, well tolerated, did not compromise surgical safety
NCT04343885 (UpFrontPSMA)	mCNPC	140	Docetaxel	Undetectable PSA rate at 12 months	N/A
NCT05079698	Oligometastatic	6	^177^Lu-PSMA-617 prior to SBRT	DLTs	N/A
NCT04443062 (Bullseye)	Oligometastatic	58	Deferred ADT	Fraction of patients with disease progression in 6 months; time to disease progression	N/A
NCT05560659 (POPSTAR II)	Oligometastatic	92	SABR	Biochemical PFS	N/A

mCRPC, metastatic castration resistant prostate cancer; RP2D, recommended phase 2 dose; ORR, objective response rate; PSA-PFS, prostate specific antigen progression free survival; DLT, drug-limiting toxicity; MTD, maximum tolerated dose; PSA, prostate specific antigen; OS, overall survival; Gy, Gray; mCNPC, metastatic castration naive prostate cancer; LN, lymph node

#### Castration-resistant prostate cancer

Immunotherapy with checkpoint inhibitors is actively being investigated in conjunction with ^177^Lu-PSMA-617 in patients with mCRPC. A phase Ib trial (NCT03805594) began studying the combination of the PD-1 inhibitor, pembrolizumab, with ^177^Lu-PSMA-617 to determine the recommended phase 2 dose (RP2D), schedule, and objective response rate (ORR) of this combination in approximately 43 patients with mCRPC [60, 61]. Similarly, a phase II trial (NCT05766371) is aiming to study whether a single dose of ^177^Lu-PSMA-617 followed by pembrolizumab was safe and provides clinical benefit in approximately 45 patients with mCRPC who have previously progressed on at least one prior ARPI (abiraterone, enzalutamide, apalutamide) [61, 63]. Another study that is looking at combining immunotherapy with ^177^Lu-PSMA-617 is the EVOLUTION study (NCT05150236). In this study, the activity and safety of ^177^Lu-PSMA-617 therapy versus ^177^Lu-PSMA-617 in combination with the CTLA-4 inhibitors, ipilimumab and nivolumab, is also being investigated in a randomized, phase II trial looking at approximately 110 patients with mCRPC. Patients will be randomized in a 2:1 ratio stratified by prior exposure to docetaxel in order to determine the PSA progression free survival (PSA-PFS) at one year [64].

Additionally, the UPLIFT study (NCT05113537) is a phase I/II trial that is uniquely investigating the use of the cyclin-dependent kinase-4 and -6 inhibitor, abemaciclib, prior to administration of ^177^Lu-PSMA-617 in order to determine the drug limiting toxicities (DLTs) and RP2D for abemaciclib given prior to ^177^Lu-PSMA-617 at each treatment cycle in approximately 30 mCRPC patients [65].

Several other drugs are also being tested in combination with ^177^Lu-PSMA-617 in the treatment of mCRPC, including cabozantinib (NCT05613894, CaboLu trial), olaparib (NCT03874884, LuPARP trial), and enzalutamide (NCT04419402, ENZA-p study). The CaboLu trial is a phase 1b dose-escalation study in which the combination of cabozantinib, a multityrosine kinase inhibitor, and ^177^Lu-PSMA-617 is being investigated to assess the rate of DLTs in the dose-escalation phase and then identifying the maximum tolerated dose (MTD) and/or recommended dose and schedule for the subsequent expansion phase. This study will look at 24 patients with mCRPC [66]. Similarly, the LuPARP study is a phase 1 trial investigating the combination of olaparib, a potent poly ADP-ribose polymerase inhibitor, and ^177^Lu-PSMA-617 to determine safety and tolerability of this combination in approximately 52 patients with mCRPC [67]. Additionally, the ENZA-p study is a randomized, stratified, 2-arm phase 2 trial that recruited 162 participants with mCRPC not previously treated with chemotherapy in order to determine the PFS of the combination of enzalutamide and ^177^Lu-PSMA-617. These patients were randomized to either enzalutamide or enzalutamide plus ^177^Lu-PSMA-617 in a 1:1 ratio [68].

Data from the ENZA-p trial showed a median PSA-PFS of 13.0 months (95% CI 11.0–17.0) in the enzalutamide plus ^177^Lu-PSMA-617 group and 7.8 months (95% CI 4.3–11.0) in the enzalutamide group (hazard ratio 0.43; 95% CI 0.29–0.63; p < 0.0001). The addition of ^177^Lu-PSMA-617 to enzalutamide improved PSA-PFS providing evidence of enhanced anticancer activity in patients with mCRPC with risk factors for early progression on enzalutamide and warrants further evaluation of the combination more broadly in metastatic prostate cancer [69].

One study is also evaluating the potential clinical benefit of combining ^177^Lu-PSMA-617 with chemotherapeutic agents. The LuCaB study (NCT05340374) is evaluating the combination of cabazitaxel and ^177^Lu-PSMA-617 in a phase I/II study to determine the MTD, DLTs, and RP2D of this combination in approximately 44 patients with mCRPC who have progressed on prior docetaxel and a second-generation androgen receptor antagonist [70].

The safety and tolerability of ^177^Lu-PSMA-617 in mCRPC patients with moderate and severe renal impairment is being examined by Novartis in a phase II study (NCT06004661). Twenty patients are expected who will be stratified to one of three cohorts based on eGFR at screen (normal, moderately reduced, or severely reduced). The effects of radiation absorption in the kidney and other at-risk organs will be determined, as well as the concentration in blood and radioactivity in the urine [71].

The purpose of the REALITY Study is to analyze the safety and efficacy of RLT targeting PSMA in men with mCRPC in everyday practice. The trial analyzed prospectively collected registry data in 254 patients with mCRPC. Primary endpoints were response to RLT, defined by changes from baseline PSA concentration, PSA-PFS, and OS. Data published in September 2021 demonstrated a greater than 50% PSA reduction in 52.0% of patients (132/254). PSA-PFS was 5.5 (95% CI 4.4–6.6) months and OS, 14.5 (95% CI 11.5–17.5) months. This large, prospectively observed “real-world” cohort of patients with late-stage mCRPC showed ^177^Lu-PSMA-617 was effective, safe, and well-tolerated [72].

#### Localized prostate cancer

The LuTectomy trial (NCT04430192) is a phase I/II trial that is seeking to evaluate the utility of ^177^Lu-PSMA-617 in an earlier disease setting. LuTectomy enrolled 20 men with high PSMA-expressing, high-risk localized or locoregional advanced prostate cancer and evaluated the dosimetry, safety, and efficacy of upfront ^177^Lu-PSMA-617 prior to radical prostatectomy with pelvic node lymph node dissection. Participants received one to two cycles of ^177^Lu-PSMA-617 followed by surgery to deduce the radiation absorbed dose in both the prostate and involved lymph nodes. The absorbed radiation doses were a median of 19.6 Gy (IQR 11.3–48.4) for the prostate, and a median of 37.9 Gy (IQR 33.1–50.1) for involved pelvic lymph nodes. The study found that up to two cycles of ^177^Lu-PSMA-617 prior to radical prostatectomy was safe, well tolerated, and did not compromise surgical safety [73].

#### Castration-naive prostate cancer

The UpFrontPSMA study (NCT04343885) is investigating the clinical benefit of the combination of ^177^Lu-PSMA-617 and docetaxel in mCNPC. UpFrontPSMA is a randomized phase 2 study enrolling approximately 140 patients with newly diagnosed mCNPC who will be randomized to either ^177^Lu-PSMA-617 followed by docetaxel or docetaxel in a 1:1 ratio. The undetectable PSA rate at 12 months is the primary outcome, while the safety of ^177^Lu-PSMA-617 followed by docetaxel compared to docetaxel alone, time to development of castration resistance between treatment arms, PSA-PFS, and rPFS will also be measured [74].

#### Oligometastatic prostate cancer

A phase I pilot study out of Memorial Sloan Kettering Cancer Center (NCT05079698) is planning to look at the administration of ^177^Lu-PSMA-617 prior to SBRT in the castration naive, oligometastatic treatment phase in an effort to prevent or delay metastasis. This study will evaluate approximately 6 patients and measure the proportion of subjects with DLTs due to ^177^Lu-PSMA-617 [75].

The Bullseye study is phase 2 prospective randomized trial (NCT04443062) that plans to investigate the efficacy of ^177^Lu-PSMA-617 in approximately 58 OMPC prior to the castration-resistant state. These patients will be randomized in a 1:1 ratio to receive either ^177^Lu-PSMA-617 or current standard of care (deferred ADT) to compare the fraction of patients that have disease progression within 6 months, and to compare the two arms for the time to disease progression [76].

The POPSTAR II study (NCT05560659) is a randomized phase II parallel cohort trial that plans to evaluate the biochemical PFS of stereotactic ablative body radiotherapy (SABR) alone and SABR plus ^177^Lu-PSMA-617 in approximately 92 OMPC patients. These patients will be split into a 1:1 ratio to either SABR alone or SABR plus 2 cycles of ^177^Lu-PSMA-617 over a period of 24 months [77].

### Phase IV ^177^Lu-PSMA-617 trials

In a phase IV, post-authorization safety study (NCT05803941), Novartis is currently investigating the long-term outcome of known or potential risks of ^177^Lu-PSMA-617. The study will seek to further characterize any other serious adverse reactions in the long-term in adults with prostate cancer who received at least one dose of ^177^Lu-PSMA-617 from interventional, phase I-IV Novartis sponsored clinical trials. The study will observe approximately 700 patients with an estimated study completion of July 21, 2033 [78].

### Final considerations

The vast exploration of ^177^Lu-PSMA-617 throughout all phases of prostate cancer treatment and promising published and anticipated results lead to optimism about the future landscape of prostate cancer treatment. However, the availability and costs associated with the treatment remain a barrier to its extensive utilization, with an expected cost of $122,489 per patient. For comparison, the estimated total treatment cost for docetaxel is $5,940 per patient, while cabazitaxel is between $18,069 and $36,137 per patient. The Canadian Agency for Drugs and Technologies in Health recommends Pluvicto be reimbursed by public drug plans for the treatment of patients with mCRPC. Future efforts will need to be directed towards improving access to care for these therapies [79].

Although the benefits of ^177^Lu-PSMA-617 therapy have been highly promoted, this treatment is not benign and adverse effects of treatment should be discussed with patients as part of the shared-decision making process prior to treatment initiation. Adverse effects include fatigue, dry mouth, nausea, anemia, changes in bowel movements (constipation or diarrhea), vomiting, thrombocytopenia, urinary tract infection, weight loss, or abdominal pain (80).

## Conclusion

RLT with ^177^Lu-PSMA-617 has demonstrated efficacy in progressive mCRPC post-ARPI and 1–2 taxane chemotherapies in patients with PSMA-avid disease through the results of the VISION trial. Preliminary results from PSMAfore in taxane-naïve mCRPC are promising which leads to anticipation of results of PSMAddition in CNPC and PSMA-DC in OMPC. While these large phase III RCTs are currently underway, numerous additional smaller phase I and II studies incorporating ^177^Lu-PSMA-617 in combination with other agents are being planned. This review article highlights the many ongoing studies of ^177^Lu-PSMA-617 which are investigating expansion of use to all phases of prostate cancer, as well as brings attention to the additional proposed adjunct uses of ^177^Lu-PSMA-617 in prostate cancer.

## Data Availability

Data sharing is not applicable to this article as no datasets were generated or analyzed during the current study.

## Abbreviations

ADT: Androgen deprivation therapy
ARPI: Androgen receptor pathway inhibitor
CNPC: Castration naïve prostate cancer
CRPC: Castration resistant prostate cancer
DLT: Drug-limiting toxicity
MFS: Metastasis-free survival
mCNPC: Metastatic castration naive prostate cancer
mCRPC: Metastatic castration resistant prostate cancer
MTD: Maximum tolerated dose
OMPC: Oligometastatic prostate cancer
ORR: Objective response rate
OS: Overall survival
PFS: Progression-free survival
PSA-PFS: Prostate specific antigen progression free survival
PSMA: Prostate specific membrane antigen
RCT: Randomized controlled trial
RLT: Radioligand therapy
RP2D: Recommended phase 2 dose
rPFS: Radiographic progression-free survival
SABR: Stereotactic ablative body radiotherapy
SBRT: Stereotactic body radiation therapy
SSE: Symptomatic skeletal event
